# Opsoclonus-Myoclonus in a Patient With Japanese Encephalitis: A Video-Based Case

**DOI:** 10.7759/cureus.23469

**Published:** 2022-03-24

**Authors:** Kumar Saurabh, Reyaz Ahmad

**Affiliations:** 1 Neurology, Tata Main Hospital, Jamshedpur, IND

**Keywords:** paraneoplastic, post-infectious, self-limiting, japanese encephalitis, opsoclonus-myoclonus

## Abstract

Japanese encephalitis is one of the most important causes of viral encephalitis in Asian countries caused by an arbovirus belonging to the flavivirus family. Complications following infection are frequent. Most common complications include dystonia, movement disorders, seizures, behavioral abnormalities, and persistent cognitive impairment. Herein, we describe a patient who after one month of being diagnosed with Japanese encephalitis developed opsoclonus-myoclonus. At a two-month follow-up, opsoclonus-myoclonus significantly improved with steroid therapy.

## Introduction

Japanese encephalitis (JE) is the most common cause of epidemic and endemic encephalitis in Asian countries. It is a member of the flavivirus family-like West Nile virus, Tick-borne encephalitis, yellow fever, dengue virus, etc. Asymptomatic infections are far more common than asymptomatic ones. The ratio of asymptomatic and mild infections to serious ones is around 250:1. However, the once symptomatic disease can cause significant mortality and morbidity [1]. Complications such as seizure, behavioral abnormalities, persistent cognitive decline, dystonia, movement disorders are common [2,3]. However, there is only one case report of JE associated with opsoclonus-myoclonus described in the literature to date [4]. Though, the association has been mentioned in a few case series of JE patients [5,6]. Opsoclonus is an involuntary conjugate, chaotic, multidirectional eye movement frequently found together with myoclonus (opsoclonus-myoclonus syndrome) [7]. Most cases of opsoclonus having known etiology are either paraneoplastic or postinfectious. In adults, the most common tumors associated with the syndrome include lung cancer, breast cancer, and gynecological malignancies [8]. Post infective cases have been described in association with infections like human immunodeficiency virus (HIV), West Nile, dengue, streptococcal infection, malaria, etc. [7]. Here, we report a case of JE who was found to have opsoclonus-myoclonus during subsequent admission after one month.

## Case presentation

A 33-year-old female patient presented with a history of headache, fever, and abnormal behavior for six days and decreased sensorium for three days. The patient had a history of hypertension, diabetes, and hypothyroidism. She is married, non-vegetarian, and without any addiction. She has studied up to matriculation. No previous history of seizure, rashes, psychiatric illness, joint pain, or rashes was present.

On examination: Temperature - 100-degree F, heart rate - 82/min, BP - 124/74 mm hg, Glasgow coma scale (GCS)-E2V2M4, neck - stiffness present, pupil - bilateral equal and reactive to light, plantar - bilateral flexor. Chest, cardiovascular and abdominal examination showed no abnormality.

**Figure 1:**
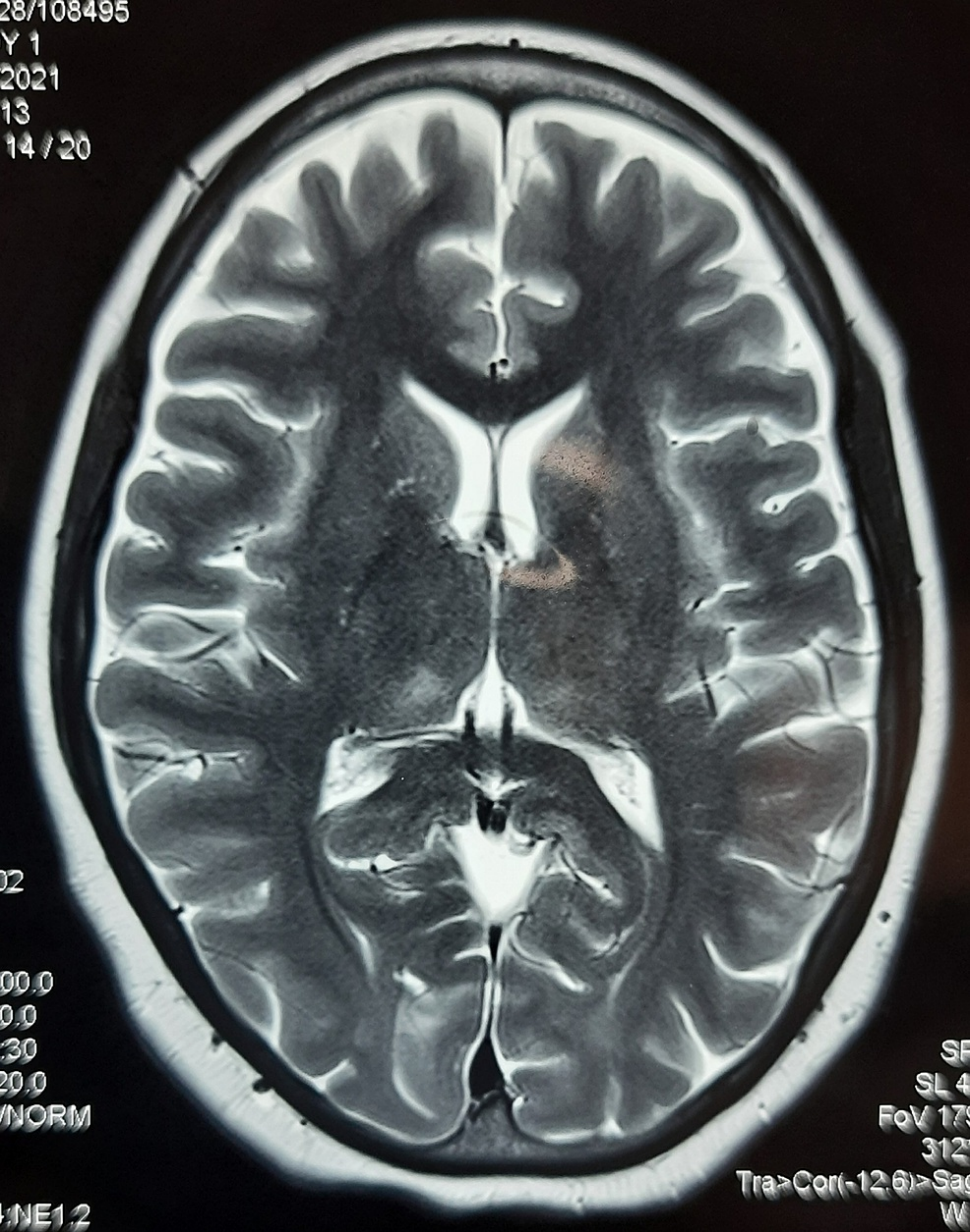
T2 MRI showing subtle bilateral thalamus involvement (right>left)

Investigations: Hg - 10.9 g/dL, white blood cells (WBC) - 12,200/cumm, platelets - 206,000/cumm, serum (Sr.) bilirubin - 1.97 mg/dL, aspartate aminotransferase (AST) - 45.5 U/L, alanine transaminase (ALT) - 47.8 U/L, Sr. creatinine - 0.78 mg/dL, blood urea - 28.3 mg/dL, Sr. sodium - 138 meq/L, Sr. potassium - 4.6 meq/L. Dengue (NS1 antigen, IgM and IgG antibodies) - negative, malaria (kit test and peripheral smear) - negative, HIV 1 and 2/hepatitis B surface antigen (HBsAg)/HCV- negative, antinuclear antibodies (ANA) - negative, COVID-19 real-time reverse transcription-polymerase chain reaction (RTPCR) - negative. Cerebrospinal fluid (CSF) examination: appearance - clear, cells - 40 (all lymphocytes), protein - 89.3 mg/dl, glucose - 59 mg/dL (corresponding blood glucose - 78 mg/dL), adenosine deaminase - 6.5 U/L, Gram stain - no organism detected, acid-fast bacilli (AFB) - absent, culture - no growth, Tuberculosis polymerase chain reaction (TB-PCR) - mycobacterium not detected, malignant cells - absent. JE IgM antibody was positive in CSF. MRI brain showed intensity changes in bilateral medial thalamus in FLAIR and T2 sequences (Figure 1).

The patient was diagnosed as a case of JE based on serological and radiological findings. During admission, she showed improvement in GCS (E4V2M5 from E2V2M4). At the time of discharge, the patient was prescribed tablet Cefuroxime (for urinary tract infection [UTI]), Pantoprazole, and Ondansetron apart from her previous medications for diabetes, hypertension, and hypothyroidism.

However, the patient was again admitted after one month. This time with complaint of involuntary movements of the eyes and head for four days. There was no history of fever or further decline in sensorium.

On examination: Heart rate - 102/min, BP - 116/88 mmHg, GCS - E4V3M5, neck - soft, pupil - bilateral equal and reactive, plantar - bilaterally flexor. Irregular, multidirectional, conjugate eye movements were noted. Jerky movement of the head, trunk, and upper limbs were present which increased on maintaining posture. Detailed neurological examination was not possible as the patient could not cooperate (Video 1).

**Video 1:** Opsoclonus-myoclonus secondary to Japanese encephalitis

Investigation: Hg - 12.6 mg/dL, WBC - 10,600/cumm, platelets - 221,000/cumm, Sr. creatinine - 0.74 mg/dL, blood urea - 32.8 mg/dL, Sr. bilirubin - 2.59 mg/dL, AST- 145.2 U/L, ALT- 330.2, Sr. sodium- 136 meq/L, Sr. potassium- 4.2 meq/L, HIV/HBSAg/HCV/hepatitis A virus (HAV) - negative. CSF examination: cells - 6 (all lymphocytes), protein - 99.4 mg/dL, glucose - 68 mg/dL (corresponding blood glucose - 96 mg/dL), Gram stain - no organism detected, culture - negative, adenosine deaminase - 2.2 U/L, AFB - not found, TB-PCR- mycobacterium not detected. Tumor markers (carcinoembryonic antigen [CEA], cancer antigen 19-9 [CA 19-9], and alpha-fetoprotein [AFP]) were negative. Non-contrast computerized tomography (NCCT) brain was within normal limits. Contrast-enhanced computed tomography (CECT) chest and abdomen revealed no abnormality other than the fibrotic area in the right postero-basal segment of the lung.

The patient was diagnosed to have post-infective opsoclonus-myoclonus secondary to previously diagnosed JE one month back. The patient's relatives opted for steroid therapy after a discussion of the pros and cons of available treatment options including cost implications. A three-day course of injection methylprednisolone was given under insulin cover. Blood pressure and sugar were diligently monitored. No improvement was noted and relatives were given the option of intravenous immune globulin (IVIg) treatment, which was denied due to affordability issues. After discharge patient was on oral steroids (methylprednisolone) with advice for daily blood pressure and glucose measurement. She was also prescribed tablet clonazepam and sodium valproate. On follow-up, after two months, the patient showed significant symptomatic improvement. Her GCS was E4V5M6. She responded to questions appropriately most of the time, though sometimes deviated from the topic of conversation (Video 2).

**Video 2:** Opsoclonus-myoclonus recovered after two months of steroid treatment

## Discussion

During the first admission, the patient was worked up for causes of febrile encephalopathy. She was diagnosed with JE based on a positive CSF JE IgM report. Also, bilateral thalami were involved in MRI which provided support for the presence of JE infection [9,10].

JE is a zoonotic disease, the primary transmission being in aquatic birds and pigs. Human beings are incidental and dead-end hosts. The disease is transmitted by Culex mosquitoes. Most cases are reported from rural and suburban areas [1]. Post-infection patients can have sequelae like behavioral issues, cognitive dysfunction, seizures, and persistent coma. Complications like abnormal movements and parkinsonism are common due to the frequent involvement of basal ganglia [2,3,11]. Acute flaccid paralysis-like state due to affection of motor neurons has also been frequently described [12]. However, only one case associated with opsoclonus-myoclonus has been described in the literature to date [4].

During the second admission, one month after discharge patient was found to have multidirectional conjugate eye movements without intersaccadic intervals. She also had an abnormal jerky movement of the head and upper limbs which increased on maintaining posture. So, she was diagnosed to have opsoclonus-myoclonus. Opsoclonus can be differentiated from nystagmus by the absence of intersaccadic intervals and from ocular flutter by the presence of multidirectional eye movements as opposed to eye movements restricted in the horizontal plane [7].

Major etiologic groups for opsoclonus-myoclonus are idiopathic, post-infectious, and paraneoplastic. The most common group idiopathic is thought to be mostly comprised of unproven prior infections. However, the temporal course of opsoclonus-myoclonus in our case was quite suggestive of a post-infectious etiology, secondary to JE infection one month back. The hallmark of the diagnostic workup for opsoclonus cases is to find or exclude paraneoplastic etiology as they require an aggressive management approach. Paraneoplastic causes were ruled out because of negative tumor markers and no findings suggestive of neoplasia in the CECT chest and abdomen. The paraneoplastic panel was not sent because of cost issues and low probability of positive results even in proven paraneoplastic cases [8]. Other rare causes like metabolic and drug-induced were also excluded because of the absence of features like hyperosmolar coma and no history of intake of drugs thought to be responsible for myoclonus like phenytoin, amitriptyline, lithium, etc.

Among postinfectious causes, association with HIV, West Nile virus, Epstein Barr virus, malaria, streptococcus, etc. have been described [7]. Breast cancer, lung cancer, and gynecologic cancer result in most paraneoplastic causes. Areas of the brain involved in disease causation have been subject to much debate. Initially, it was thought that the underlying cause was an imbalance between burst and omnipause neurons in the brainstem controlling saccadic output. But now the focus has shifted to the role of disinhibition of Purkinje cells in disease pathogenesis [7]. Autoimmunity is thought to be central in disease pathogenicity. Antibodies generated due to molecular mimicry between tumor or infectious agents and the brain cells can result in disease manifestation. The finding of oligoclonal bands, BAFF (B-cell activating factor), and altered cytokine profile in CSF are all suggestive of autoimmunity [13-16].

More research is required to find the best treatment regimen as very few randomized controlled trials and other studies are available due to disease rarity. It is generally agreed that for mild cases symptomatic therapy like clonazepam and antiepileptics may be enough. However, moderate to severe disease will require immunomodulators. Generally, steroids are started first because of easy availability and cost issues. One research showed good efficacy of combination IVIg therapy [17]. Rituximab was also found to be effective in various studies with one study showing prolonged suppression of CSF B-cells even one year after receiving the drug [18]. Once disease control is achieved drug tapering should be slow, more so in paraneoplastic cases to avoid relapses that can be difficult to treat resulting in significant sequelae. Ideally, a state of NEDA (no evidence of disease activity) should be achieved before drug tapering. CSF parameters like cytokine profile, BAFF, and alteration of B cell subset have the potential to be used as biomarkers to guide drug tapering in the future [19]. Our case received steroids apart from symptomatic therapy and had almost complete resolution at two months follow-up. There was no relapse one month after tapering off steroids. This is in line with various case series showing a better prognosis of post-infectious cases as compared to paraneoplastic ones. Postinfectious cases are mostly monophasic with the self-limiting course and reports of poor outcome and relapse are rare [8,20]. They may improve spontaneously but immunomodulators can hasten recovery. However rare postinfectious cases with severe presentation and relapses may need multimodal immunotherapy.

## Conclusions

JE infection can result in rare complications like opsoclonus-myoclonus as demonstrated in our case. Upon presentation, opsoclonus should be distinguished from other eye movement disorders like nystagmus and ocular flutter. It is prudent to exclude paraneoplastic causes as they are associated with worse outcomes and usually require higher immunomodulators apart from a specific therapy. Our case highlighted the fact that post-infective cases of opsoclonus-myoclonus may have good recovery without the use of higher or combinational immunomodulators.
